# The safety and feasibility of a pilot randomized clinical trial using combined exercise and neurostimulation for post-stroke pain: the EXERT-Stroke study

**DOI:** 10.3389/fneur.2025.1524004

**Published:** 2025-04-24

**Authors:** Chen Lin, Charity J. Morgan, E. Lane Schlitz Fortenberry, X. Michelle Androulakis, Keith McGregor

**Affiliations:** ^1^Birmingham VA Medical Center, Birmingham, AL, United States; ^2^Department of Neurology, The University of Alabama at Birmingham, Birmingham, AL, United States; ^3^Departments of Biostatistics, The University of Alabama at Birmingham, Birmingham, AL, United States; ^4^Charleston VA Medical Center, Charleston, SC, United States; ^5^Departments of Clinical and Diagnostic Sciences, The University of Alabama at Birmingham, Birmingham, AL, United States

**Keywords:** stroke, post-stroke pain, post-stroke headaches, clinical trial, neuromodulation, exercise stroke, exercise, neurostimulation

## Abstract

**Background:**

Chronic pain after stroke can occur between 10 and 50% of stroke survivors. Post-stroke pain (PSP) can lead to further complications in a stroke survivor’s recovery. PSP is caused by the stroke itself and produces moderate or severe pain. It can manifest as new onset or worsening of prior headaches.

**Methods:**

EXERT-Stroke was a feasibility pilot 2-arm randomized sham-controlled, double-blind trial at a single center over a 30-day intervention period, followed by a month follow-up. Patients were recruited for this study from July 2022 through June 2024 at the Veterans Hospital. The study protocol was approved by the local institutional review board. The trial was registered with ClinicalTrials.gov (NCT04672044). All potential participants were screened for safety with a graded exercise stress test before randomization. Participants were randomized (1:1) to either active repetitive transcranial magnetic stimulation (rTMS) or sham rTMS. Both arms received the same exercise protocol. The intervention protocol consisted of 10 sessions over a 30-day period of rTMS (sham vs. active) + exercise, where rTMS was delivered prior to each exercise session on the same day. RTMS was aimed at the M1 of the contra-lesional hemisphere. Exercise was delivered on a recumbent bicycle targeting a participant’s heart rate reserve. Primary outcomes were intervention feasibility (attendance and tolerance) and safety (adverse events).

**Results:**

Of those consented, one participant was a screen failure, and nine participants were randomized. The average age was 62 years old, 22.2% were female, and 44.4% were Black. For feasibility, five (55.5%) participants were randomized to active rTMS and four (44.4%) were randomized to sham rTMS. Four of the five (80%) active rTMS and two of the four (50.0%) of the sham rTMS completed the final assessment, suggesting that there was no association between treatment assignment and likelihood of completing the study. Importantly, there were no serious adverse events.

**Conclusion:**

This is the first feasibility trial to investigate paired intervention of exercise and rTMS in patients with post-stroke pain. The trial found that the intervention had few safety issues. There was overall positive feedback from participants.

**Clinical trial registration:**

https://clinicaltrials.gov/, identifier NCT04672044.

## Introduction

Chronic pain after stroke can occur between 10 and 50% of stroke survivors (1, 2). Post-stroke pain (PSP) can lead to further complications in a stroke survivor’s recovery including worsening depression, causing cognitive dysfunction, and even increasing suicidality rates (1, 3). PSP is caused by the stroke itself and produces moderate or severe pain. It can manifest as new onset or worsening of prior headaches and/or somatic pain in the topographic region of the brain affected by the stroke. In 2018, persistent post-stroke headache, the pain syndrome focused in this proposal, was defined in the International Classification of Headache Disorders, 3rd ed. (4). While acute headaches after stroke resolve within 3 months, persistent post-stroke headache have been defined to last >3 months and no other pain diagnosis better explains the symptoms. Since post-stroke headaches is a relatively new diagnosis, there are no specific treatment guidelines on this significant issue, particularly as migraines can commonly impact Veterans (5). While the mechanisms are poorly understood, persistent post-stroke headaches are more frequent and more severe than acute stroke-related headaches (6). Current guidelines of post-stroke pain generally leads to pharmacologic treatments including some clinical management with opioids, which can lead to addiction issues (7, 8). There are even fewer nonpharmacologic options known, but both exercise and neurostimulation have demonstrated some potential to alleviate pain (7, 8).

In stroke survivors, exercise has improved symptoms such as mobility, fatigue, and quality of life (9, 10). A statement by the American Heart/Stroke Association recommended that exercise should be incorporated into the management of stroke survivors, noting the evidence strongly support the benefits early after stroke (11). For survivors, optimal exercise programs have begun at least a month after stroke and durations that last 1–3 months (12). With exercise protocols, the goal is target-based dosing using target heart rate reserve and target heart rate dosing (13, 14). Thus, repetition and time spent exercising matters less but reaching a certain physiologic state demonstrates reaching a sustainable target benefit from exercise (13, 14).

Repetitive transcranial magnetic stimulation (rTMS) uses noninvasive electromagnetic induction for cortical neurostimulation. The use of rTMS has been explored in several trials for patients with neuropathic pain and those with chronic PSP. The primary motor cortex has been used as the primary site for stimulation in several studies, including multicenter double-blind sham-controlled randomized clinical trials, examining the efficacy of rTMS for chronic PSP (15–17). Studies suggest that neurostimulation of the motor cortex modulates interconnected neural structures modulate pain pathways (2, 18). A commissioned panel of experts to establish rTMS guidelines on therapeutic use gave rTMS of the contralateral primary motor cortex a Level A recommendation (19). The panel concluded the evidence for rTMS as a definite analgesic effect with few safety issues (19). However, the effects of the treatment are transient, usually lasting only a few hours to days (20).

For rTMS to be a practical therapy for chronic PSP, more sustained efficacy would need to be demonstrated (2). In rTMS studies for stroke rehabilitation, neurostimulation was delivered prior to physical activity (21, 22). This additional neurostimulation paired with physical activity was thought to help sustain motor recovery from physical activity. The primary aim of the EXERcise and repetitive Transcranial magnetic stimulation in post Stroke pain (EXERT-Stroke) trial was to investigate the safety and feasibility of our combined intervention protocol using moderate-intensity interval training (MIIT) exercise and repetitive transcranial magnetic stimulation in patients with persistent post-stroke headaches. Specific objectives included: determining how many patients were needed to be pre-screened to reach our target consent number, identifying retainment issues for those randomized, and if any adverse events could occur during our combined intervention.

## Methods

EXERT-Stroke was a pilot 2-arm randomized sham-controlled, double-blind trial at a single center over a 30-day intervention period, followed by a month follow-up. Patients were recruited for this study from July 2022 through June 2024 in the Neurology Clinic at the Birmingham VA Medical Center. This study was conducted in accordance with the 1964 Helsinki declaration. The study protocol was approved by the local institutional review board (ID# 1600274). All participants provided written informed consent before participating in any study procedures. The trial was registered with ClinicalTrials.gov (NCT04672044). CONSORT checklist is included in supplemental files. The full study protocol is available at reasonable request to the corresponding author. No important changes to methods were made after trial commencement. No changes to the pilot trial assessments or measurements were made after trial commencement.

Participants were eligible if they were (1) Male or female Veteran of US military ≥19 years of age; signed informed consent; (2) Minimum of 3-months since time of stroke and medically stable; (3) New or Worsened headache in close temporal relationship with stroke that has persisted for >3 months after stabilization of the stroke; (4) Ability to walk or tolerate recumbent cycle ergometry for 10 min without assistance; (5) Stable pain medication regimen for ≥1 month prior to study; (6) Females of child-bearing potential (i.e., not postmenopausal or surgically sterile) must be using a medically acceptable method of birth control and should not be pregnant nor have plans for pregnancy or breastfeeding during the study; (7) Completed diagnostic, maximal graded exercise test including 12-lead ECG and indirect calorimetry (i.e., oxygen uptake, minute ventilation, respiratory exchange ratio, etc.), and cleared for participation by an nurse research coordinator; and (8) Minimum pain intensity of 30 on the Mechanical Visual Analogue Scale on average with pain symptoms.

Participants were excluded for the following: (1) Moderate to severe cognitive impairment (Montreal Cognitive Assessment score <16/30); (2) Pre-stroke modified Rankin >2; (3) History of seizures; (4) Presence of any standard TMS or MRI contraindications (see human subjects); (5) Current diagnosis of DSM-5-defined bipolar disorder I, schizophrenia, schizoaffective disorder, or obsessive-compulsive disorder; (6) Diagnosis of moderate or severe substance use disorder (except for caffeine and nicotine) during the preceding 3 months (Participants must agree to abstain from illicit drugs during the study); (7) Increased risk of suicide that necessitates inpatient treatment or warrants additional therapy excluded by the protocol; and/or intensity of suicidal ideation (Type 4 or Type 5) or any suicidal behavior in the past 3 months on Columbia Suicide Severity Rating Scale (C-SSRS); (8) Veterans litigating for compensation from a psychiatric disorder related to their VA service-connected disability; (9) Current enrollment in another intervention trial for pain or stroke; (10) Persons imprisoned, of minor age, diagnosed with terminal illness, or require surrogate for consent; (11) Fails baseline exercise screening activities; (12) Headaches that were better accounted for by another diagnosis other than post-stroke headache; (13) Is unable to reliably attend intervention sessions, i.e., planning to move, transportation issues; and (14) Neurological disorder pre- or post- stroke affecting subject’s ability to follow study directions.

### Screening and randomization

The screening procedure included informed consent, vital signs, demographics, medication history, neurologic and psychiatric diagnostic evaluation, imaging studies, and medical history. Participants return for the baseline visit (within 2 to 30 days of screening). All potential participants passed a graded exercise stress test before randomization at the baseline visit. The test included maximal graded exercise test including 12-lead ECG and clearance for participation was performed by a Cardiologist after the test.

After screening, consent, and baseline activities, participants were randomized (1:1) to either active rTMS or sham rTMS. Both arms received the same exercise protocol. The randomization scheme was developed using random permuted blocks with block sizes 2, 4 and 6 by an unblinded study statistician. Sealed envelopes marked with the subject number on the outside were used to deliver the treatment assignment to the research technologist who delivered the TMS intervention. The envelopes were opened by the technologist and the technologist and study statistician were the only study personnel who were unblinded to the active vs. sham assignment. All investigators and participants were blind to the treatment group. Based on available study resources and budget, it was calculated that we would be able to screen and consent up to ten participants, before randomization. Of these ten screened participants, we assessed the feasibility of our screening criteria by capturing the number of screen and pre-screen failures to help with future trial design. The remaining participants that were randomized and participated in the intervention were assessed for safety, tolerability, and adherence to the intervention. There were no prespecified criteria to judge for how to proceed with future definitive trial.

### Intervention

After screening and randomization, the intervention protocol consisted of 10 sessions of rTMS (sham vs. active) + exercise, where rTMS was delivered prior to each exercise session on the same day, up to 1 h apart. These 10 intervention sessions occurred over a 30-day period.

### rTMS protocol

Both rTMS groups, active and sham, were conducted identically. Resting motor threshold (RMT) was defined as the minimal intensity of stimulation while muscle (APB) is relaxed to elicit motor evoked potential of more than 50 mV in amplitude. rTMS was performed with a figure-of-eight shaped coil centered over the RMT target, M1 of the contra-lesional hemisphere. If patient had bilateral hemisphere infarcts, then the more symptomatic headache side was targeted. Stimulation was delivered using the Magstim magnetic stimulator (The Magstim Co., Whitland, United Kingdom) with a figure-of-eight shaped coil (70-mm Double Coil, #9925-00, Magstim). rTMS was performed using the Super-Rapid Magstim magnetic stimulator with a figure-of-eight shaped coil centered over the RMT target. Each rTMS session consisted of a series of 30 trains of 10 s in duration (30 s intertrain interval) at a stimulation rate of 10 Hz (3,000 pulses) and an intensity set at 90% of RMT. The frequency and duration were chosen because significant pain relief were obtained in prior studies in chronic neuropathic pain (15, 18, 23). A sham figure-of-eight coil (#1730-23-00, Magstim) was used for the control group. The sham coil looks the same as the active coil and produces discharge noise and slight sensory stimulation of the scalp but does not induce any substantial current in the cortical tissue.

### Exercise sessions

Exercise sessions were performed with trained individuals with experience in the local rehabilitation gym. Sessions were conducted on a stationary recumbent bicycle (Manufacturer: Spirit Fitness, Model number XBR95). Each exercise session began with seated resting assessments for blood pressure, heart rate reserve and heart rate (HR). Vitals were collected pre-exercise and post-exercise. Heart rate reserve was calculated using the Karvonen formula: pt’s maximum HR – resting HR. Max heart rate = 220-age. Our exercise target was based on the following: Heart rate reserve x %target + resting HR. Each session began with a 10 min warm-up at 30% exercise target, followed by the 25 min or moderate intensity and ends with a 10 min cool-down at 30% exercise target. The exercise prescription used the percentage of the exercise target during the exercise test and ensure appropriate heart rate zones. Performance was encouraged.

#### Moderate intensity interval training

After the 10 min warm-up, MIIT consisted of repeated 1 min moderate intensity bursts (“on” interval) alternated with 1 min interval recovery (“off” interval) for 25 min. The “on” interval began at 60% of peak watts (range: 55–65%) followed by the “off” interval at 45% of peak watts (range: 40–50%). The average HR for the “on” intervals should not exceed 60% HR reserve. There was 13 min of “on” and 12 min of “off” interval exercise. Cool-down commenced after the last interval. Stopping rules: Exercise was stopped due to inappropriate HR or BP responses, or at the participant’s discretion. In cases where the participant reported intolerance or undue fatigue, workload adjustments (e.g., 10% reduction) were made (and recorded) to encourage completion of the exercise session. Participant monitoring: Each individual session during supervised exercise training was monitored closely by a research nurse or exercise scientist.

### Data collection

Primary outcomes were intervention feasibility safety (adverse events [AEs]), and acceptability (assessed via patient questioning). Serious adverse events were defined as any events that led to significant clinical intervention, hospitalization, or death. Significant clinical intervention included treatment for seizures related to r TMS. Any adverse event included discomfort from the interventions including pain, irritation from rTMS and self-reported pain leading to stopping of exercise. Safety data was collected at each visit for the intervention. Feasibility was determined by recruitment rate, consent and refusal rates, and attrition and retention rates. Acceptability was performed via direct questioning with patients on their likes, dislikes, and ideas for suggested improvements at the end of the study. All data was collected by trained study coordinators (two throughout the study duration).

### Statistical analysis

Data were summarized using means and standard deviations for continuous outcomes and counts and proportions for categorical outcomes. As this was a small pilot trial whose primary objective was to demonstrate intervention feasibility, we did not plan to perform any formal statistical hypothesis tests. As a result, a formal power calculation was not performed in order to determine the sample size. Instead, we selected our target sample size based on the desired precision for confidence intervals and the suggestions in, which recommends a sample size of 12 subjects per arm for pilot and feasibility studies. No interim analyses were conducted (24). The trial would be stopped if significant serious events occurred to 20% or more of the participants receiving intervention. For acceptability, patient responses to direct questioning were qualitatively recorded.

## Results

### Enrollment, follow-up, and demographics

**Figure 1:**
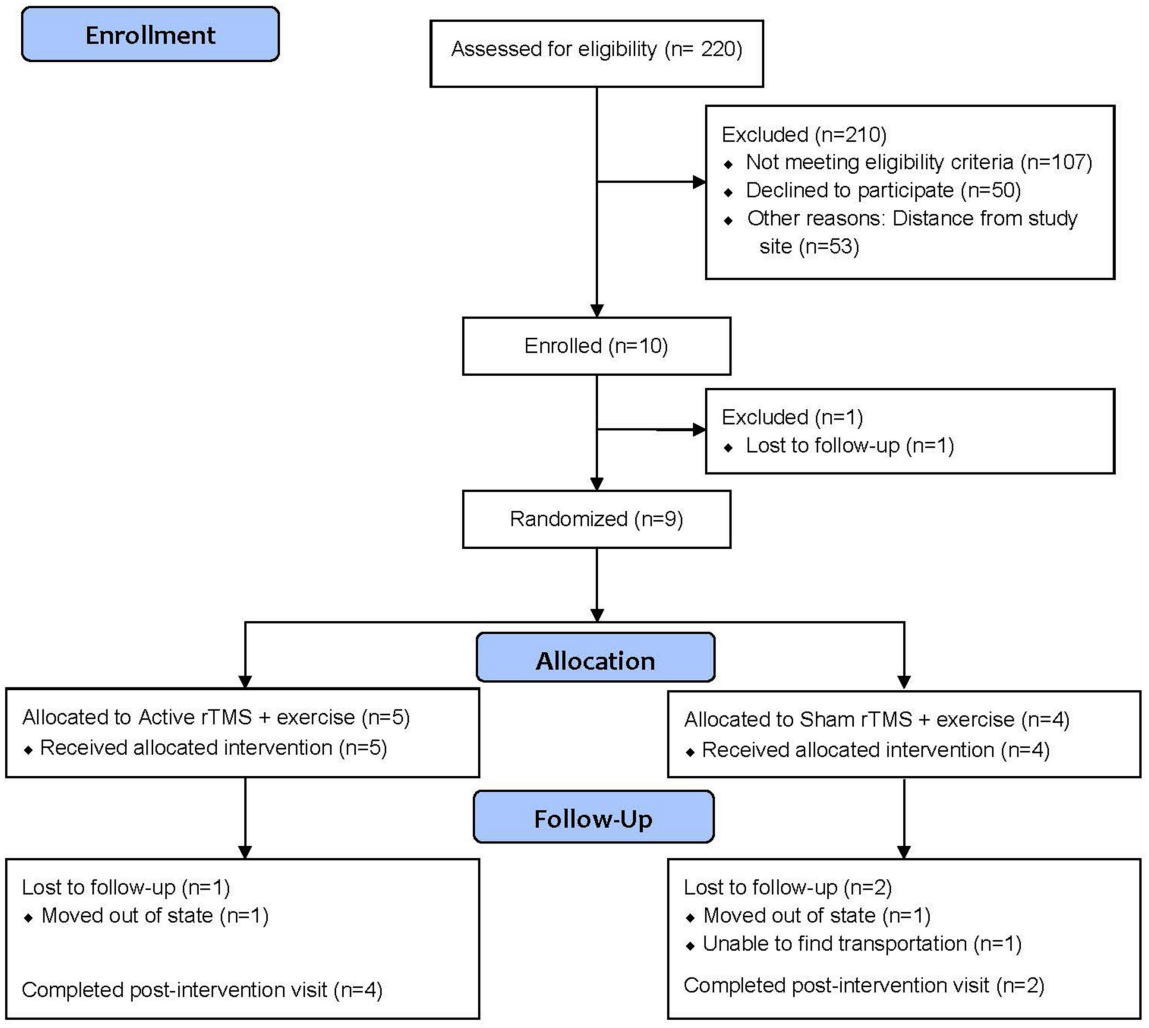
Study flowchart. rTMS, repetitive transcranial magnetic stimulation.

From July 2022 to June 2024, we pre-screened 220 patients from the Birmingham VA Medical Center neurology clinic. Our enrollment summary is detailed in Figure 1, including reasons for pre-screen and screen failure. Ninety-five percent (*n* = 210) of those assessed for eligibility did not meet eligibility criteria (*n* = 107), declined participation (*n* = 50), or were unable to participate due to distance from the study site (*n* = 53) Ten participants were consented and enrolled into the pilot trial. Of those consented, one participant was lost to follow-up before randomization, and nine participants were randomized. All randomized participants completed the baseline assessment. Of those, six (66.7%) completed the final follow-up evaluation. Participant demographics are provided in Table 1. The average age was 62 years old, 22.2% were female, and 44.4% were Black.

**Table 1:** Demographics.

Characteristic	All participants (*N* = 9)
Age, mean (SD)	62.0 (8.0)
Sex, *N* (%)	
Female	2 (22.2%)
Male	7 (77.8%)
Race, *N* (%)	
Black/African American	4 (44.4%)
White	4 (44.4%)
Other	1 (11.1%)
Ethnicity, *N* (%)	
Not reported	2 (22.2%)
Non-Hispanic/Latino	7 (77.8%)

### Feasibility

During the 24 months of the enrollment period, ten participants were enrolled, giving a recruitment rate of 0.42 participants per month. Of the 220 patients assessed for eligibility, 107 did not meet the eligibility criteria; the remaining 113 patients were used to calculate the consent and refusal rates. Ten (8.8%) of the eligible patients consented to enrolling in the trial and 103 (91.1%) refused enrollment. We note, however, that 53 (51.5%) of those refusing enrollment cited distance from the study site as their reason for declining.

Five (55.5%) participants were randomized to active rTMS and four (44.4%) were randomized to sham rTMS. Six of the nine randomized participants completed the final assessment, giving a retention rate of 66.7%. Four of the five (80%) active rTMS and two of the four (50.0%) of the sham rTMS completed the final assessment, suggesting that there was no association between treatment assignment and likelihood of completing the study. Three participants dropped out of the study early, giving an attrition rate of 33.3%. Two of these participants had to move out of state for caregiving reasons and the other one was lost to follow-up.

The mean (SD) number of exercises sessions completed by the participants was 6.1 (4.3). Notably, five (55.5%) participants completed at least eight of the ten exercise sessions, with four of these completing all ten sessions. Only one (11.1%) participant did not complete any of the exercise sessions; the remaining three (33.3%) participants completed either two or three sessions.

### Safety

No serious adverse events were reported during or following the study. One patient reported mild discomfort with the rTMS: discomfort reported with rTMS and chair positioning. This patient was in the active rTMS group. No participants reported any adverse events with exercise.

### Tolerability

One of the participants that completed the baseline, and final follow-ups could not complete all the intervention sessions due to an unrelated motor vehicle accident that prohibited their transportation. Otherwise, there were no other limitations to tolerability in either the sham or the active arm of rTMS. There were no tolerability concerns with the moderate intensity interval exercise protocol. Patients generally noted that the screening cardiac test, because it was done upright on the treadmill, was more intense than the exercise on the recumbent bicycle. Overall, feedback from participants supported both feasibility and tolerability of our combined intervention. In Table 2, we included specific comments that could inform us on future study design.

**Table 2:** Examples of comments from participants on the likes and dislikes of using rTMS and exercise.

Likes	Dislikes	Suggested improvements
Liked that they gathered information about my brain through an MRI to help understand why I am having headaches	Disliked the long drive to the VA	Facility closer to home
Liked that I became more knowledgeable about what was going on with my health	Travel time	Options for home treatments
Legs felt stronger after the exercising	Having to go to different areas for rTMS and exercise	Offer more solutions or options for reasons for the headaches
More strenuous exercise than would do on my own which is good	Discomfort with TMS and positioning of chair	Easier transportation issues
Headaches lessoned after TMS		
Enjoyed the experience		
Experience with study staff		
Helpful to learn some things and if my participating helps somebody else all the better.		
Get in better shape		

## Discussion

In this pilot trial examining the safety and feasibility of combined active rTMS+exercise compared to sham rTMS+exercise, we found that the combined intervention appeared feasible and safe. To our knowledge, this is the first study to examine this type of combined intervention for people with PSP, specifically with persistent post-stroke headaches. Several lessons were learned during the trial. The screening practices and using a combined intervention requiring frequent in-person visits were likely the aspects most challenging for both study personnel and potential participants. Although we did not meet the target sample size due to lower-than-expected recruitment rate, we were still able to demonstrate the feasibility and safety of the intervention. The estimated rates of recruitment, consent, refusal, retention, and attrition may be helpful in designing future larger trials. As we did not conduct or plan to conduct any formal statistical tests, the reduced power from a smaller sample size is not an issue here.

One of the primary issues the participants faced was distance and travel. Of the pre-screened potential participants, fifty-three declined specifically due to distance of their residing location to our study center. Of the three patients that dropped-out, two had to move out of state unexpectedly for various reasons and could not come back to our center. One participant that did not complete all their intervention sessions but could come back to her final follow-up was also due to transportation issues, having lost their car in a motor vehicle accident. To make this intervention more accessible and increase feasibility, the intervention would need to meet the patients where they are, either in a closer facility or even at home as some participants noted. Specifically, telehealth has been trialed in stroke rehabilitation (7). Primarily, telerehabilitation has been performed in patients after stroke with speech and motor deficits (25, 26). Interesting, there are very few combined interventions delivered via telehealth for patients with stroke and none, from our review, specifically addressing post-stroke pain and headaches.

Potential future directions could include combining a home-based exercise protocol with a more portable neurostimulation delivery system. A variety of devices have been extensively studied for headache disorders. None have been specifically investigated for an indication of post-stroke headaches, but most are generally considered safe in patients with stroke. One of those that could be used in home-based protocols are transcranial direct current stimulation (tDCS) devices. The growing body of evidence supports use of tDCS for chronic pain and headaches (27, 28). Remotely supervised tDCS has been used in over 6,000 at-home tDCS sessions, with no severe adverse events or emergencies having been reported to date (29). Combining remote tDCS with other tasks may further improve clinical outcome by priming neural pathways and facilitating neuroplasticity (30). Furthermore, feasibility and preliminary efficacy of combined tDCS with mindfulness in Veterans have been demonstrated in another secondary headache disorder-persistent post-traumatic headache attributed to traumatic brain injury (29). Adherence rate among Veterans who received 20 sessions of remote tDCS via VA telehealth platform was more than 80% in this study. From our knowledge, there has been only one randomized clinical trial combining our two types of interventions, but this was not delivered remotely nor was it done in patients with stroke (31). Separately both exercise (32, 33) and tDCS (34) have been shown to have different benefits in patients with stroke. Combining these two interventions, as we did with rTMS and MIIT exercise, and delivered remotely could increase both access and participation in future studies for PSP and headaches.

The combination of exercise with exogenous neurostimulation has yielded efficacy in studies involving chronic pain of various etiology including headache. Physical exercise as an adjuvant therapy to the application of rTMS has shown large effect sizes on osteopathic and neurological pain, particularly with stimulation of primary motor cortex (19, 35, 36). The upregulation of proprioception and kinesthetic sense associated with exercise may act synergistically with focal increases in cortical excitability (37). Higher excitability may induce neuroplastic cortical mechanisms affording a time window for selective modification of pain interoception (sense of body state). The 10 Hz rTMS to M1 further increases cortical activity and previous research has shown M1 stimulation may affect downstream brain regions including anterior cingulate, and periaqueductal gray associated with head and upper limb pain (19, 36). Additionally, exercise increases neurochemical release (brain-derived neurotrophic factor and endogenous opioids) affording increased analgesia through adaptive post-release neuroplastic processes. Additional research beyond this pilot is warranted to examine the post-exercise neurochemical changes.

Our study’s use of interval-based training is noteworthy and continues a growing literature on the effectiveness of this training approach. The periodic bouts of varied increased effort is more effective than steady continuous training in previous studies with older adults (38). As above, adaptive plasticity may be selectively induced through titration of challenge to the individual to provide enough of a stimulus to require an adaptive response. The present findings extend previous work in aging and chronic stroke.

There are limited therapeutic options for headache in patients with a history of stroke. There is currently no FDA approved treatment for post-stroke headache. Traditional headache medications such as Triptans, ergots, and even nonsteroidal anti-inflammatory drugs, are contraindicated in patients with a history of stroke due to increased risks of severe adverse effects. Newer generations of treatments for headache and migraines are also becoming more popular but have potential risks. Safety data is limited on these therapies such as gepants which are Calcitonin Gene-Related Peptide receptor antagonists. Safety warnings for this class of medications include new onset or worsening of existing hypertension. In mouse models, these receptor antagonists have been shown to worsen cerebral ischemia (39). Another newer class of headache therapies are the ditans, which can have low tolerability due to memory and concentration impairment, fatigue, nausea, driving restrictions, addiction potential, and carry a schedule V controlled substance designation. Several of these concerns make prescribing this class of drugs a challenge in our target Veteran population. From our participants’ qualitative comments, Veterans are interested and sometimes prefer non-pharmacological approaches and neuromodulation to their headache management. Therefore, there is an unmet clinical need for using novel non-pharmacological neuromodulation for this debilitating PSP and headache disorder.

While we conducted the first randomized clinical trial in patients with post-stroke headache and pain using a combined intervention, there were several limitations. While our preliminary findings suggest the intervention had few safety issues and was generally well-received, conclusions are limited by the reduced sample size, which also precluded a formal intention to treat analysis (40). Second, our population consisted of only Veterans recruited from a VA Medical Center, the current generalizability is limited. However, we did screen out traumatic injuries as cause for headaches, and set a strict criteria for post-stroke headaches definition based on the International Classification of Headache Disorders, 3rd ed. criteria (4). Because the study also recruited Veterans, concomitant mental health conditions and fatigue are prominent, particularly in those with stroke (41, 42). While we excluded patients with significant psychiatric conditions that could preclude them from tolerating our intervention, there would be room in future studies to examine potential concurrent mental health symptom changes as part our protocol. With these limitations, a larger efficacy study in Veterans and the civilian populations are needed. These studies should make it more practical and accessible for participants but should still be powered for efficacy using a control group such as what we did with the sham rTMS group versus an active comparator group.

## Conclusion

This is the first feasibility trial to investigate paired intervention of exercise and rTMS in patients with post-stroke pain. The trial found that the intervention had few safety issues. There was overall positive feedback from participants.

## Data Availability

The datasets presented in this article are not readily available because the study was funded by the Department of Veteran Affairs. Therefore, they also control the data, and data requests would go through them as well. For more information and requests to access the datasets should be directed to https://www.hsrd.research.va.gov/for_researchers/cyber_seminars/archives/video_archive.cfm?SessionID=1205.
